# Review on risk factors, classification, and treatment of sternal wound infection

**DOI:** 10.1186/s13019-023-02228-y

**Published:** 2023-05-19

**Authors:** Yaoyao Song, Wanli Chu, Jiachen Sun, Xinzhu Liu, Hongjuan Zhu, Hongli Yu, Chuan’an Shen

**Affiliations:** Senior Department of Burns and Plastic Surgery, Fourth Medical Center of Chinese PLA General Hospital, No. 51, Fucheng Road, Haidian District, Beijing, 100048 China

**Keywords:** Sternal wound infection, Risk factors, Classification, Wound reconstruction strategies

## Abstract

Sternal wound infection (SWI) is the most common complication of the median sternal incision. The treatment time is long, and the reconstruction is difficult, which causes challenges for surgeons. Plastic surgeons were often involved too late in such clinical scenarios when previous empirical treatments failed and the wound damage was relatively serious. Accurate diagnosis and risk factors against sternal wound infection need to be in focus. Classification of different types of sternotomy complications post-cardiac surgery is important for specific categorization and management. Not familiar with this kind of special and complex wound, objectively increasing the difficulty of wound reconstruction. The purpose of this comprehensive review is to review the literature, introduce various SWI risk factors related to wound nonunion, various classification characteristics, advantages and disadvantages of various wound reconstruction strategies, to help clinicians understand the pathophysiological characteristics of the disease and choose a better treatment method.

## Introduction

As early as 1957, the median sternal incision was first reported for open-heart surgery, which has become the standard surgical approach for thoracic and cardiac surgery [1]. As one of the common complications, SWI has been gradually taken seriously. It has been reported that the incidence rate of SWI is 1%~5%. Severe cases can lead to heart, lung, and kidney-related organ failure and even death. The mortality rate is 10%~30%^2^. In the United States, about 700,000 open heart operations are performed every year, and nearly 8300 patients develop sternal incision infections [3]. The long-term non-healing of the wound, repeated debridement, and failure of wound closure bring huge mental pressure to the patients, affect the quality of life, prolong the hospital stay, bring high costs, and increase the economic burden of the patients. There are many risk factors for sternotomy complications, including age, body mass index, smoking, and complications such as low immunity, diabetes, radiation, reoperation, and chronic lung and kidney diseases [4]. SWI can be classified as the surgical incision infection, mainly occurring within one month after cardiac surgery. It can be divided into two types according to the level and depth of the infection. Superficial sternal wound infection (SSWI) only accumulates on the skin, subcutaneous tissue, and deep fascia, while deep sternal wound infection (DSWI) can affect muscle tissue, sternum, sub sternum, and mediastinum. Other scholars believe that SSWI accumulates skin and subcutaneous tissue, and chest muscle tissue, while DSWI is mainly a mediastinal infection [5]. It should be noted that the mortality of mediastinal infection is very high, which still remains as high as 3% ~ 35%^6^. SSWI can be corrected by having debridement and direct closure of the wound edge. DSWI refers to the correction of complex defects, and there are various methods including greater omentum, tissue flap, platelet-rich plasma (PRP), and so on. There is no standard treatment method currently.

## Diagnostic procedures

### Diagnosis

Sternal wound infection, also known as post-thoracotomy infection nonunion or median sternal incision infection, refers to the infection of soft tissue and/or sternum, ribs, costal cartilage, and mediastinum under the influence of local or systemic factors after median sternal incision. DSWI wounds are limited to the deep fascia and are easier to diagnose. Whereas DSWI, according to the Centers for Disease Control and Prevention (CDC), DSWI can be confirmed by the presence of skin redness and swelling, increased skin temperature, fever (> 38℃), pain, and other infectious symptoms accompanied by purulent secretions, supplemented by etiological and pathological evidence after the median thoracic incision in thoracic and cardiac surgery. According to the clinical manifestation, with X-ray, CT, and other medical film examinations, it is found that mediastinal widening, mediastinal gas-liquid level, mediastinal emphysema, pleural effusion, and wire displacement have value in early DSWI diagnosis, and CT shows a better effect [7–11].

### Auxiliary diagnosis

In clinical practice, we found that chest X-ray films are not helpful for the diagnosis of mediastinitis. Because there are many metal internal fixators in the body of patients undergoing cardiac surgery, MRI, which has better imaging of soft tissue, cannot be used, after admission, each patient should preferably undergo a chest CT examination before debridement. Determine the “stability of the sternum” to facilitate more thorough debridement and prepare for subsequent wound closure treatment. However, a CT examination is also insufficient. It is reported that its ability to identify costochondritis is very limited. Cardiac surgery patients often have metal internal fixators, and MRI, which is better for soft tissue filming, cannot be used. But the studies have shown that the sensitivity and specificity of PET/CT were higher than that of CT. In the CT diagnosis of patients with costochondritis complicated by DSWI, the sensitivity and specificity of senior radiologists were 87.6% and 56.9%, which were far less than that of PET/CT. PET/CT can effectively reduce the dependence of clinicians on film readers and reduce the occurrence of missed diagnoses [12–14]. The bacterial culture of wound secretions is a necessary indicator for formulating anti-infection plans, wound classification, and formulating treatment plans, but there are occasional false negatives. Nick et al. using classical microbial culture, FISH combined with molecular nucleic acid amplification technology (FISHseq) to analyze specimens from 12 patients, found that microbial biofilms did not always exist in DSWI wounds, but the microorganisms were “plaque-like” in the tissue distributed Therefore, deep excision of the wound must be carried out to control the infection [15]. It is suggested that culture analysis should be carried out on at least two wound samples from different locations, and FISHseq should be used for additional molecular biological analysis when it is difficult to explain.

## Risk factors

Risk factors for sternotomy wound complications include: patient-related risk factors were obesity, advanced age, active smoking, diabetes, coronary artery disease, low ejection fraction, steroid treatment, chronic infections, innutrition, kidney disease, and chronic lung disease. Procedure-related risk factors were inadequate skin preparation, use of bone wax, emergency operation, nonskeletonized (pedicled) or bilateral harvesting of the internal mammary artery (IMA), blood product infusion, prolonged operative time and perfusion time, sternal rewiring, postoperative bleeding, use of an intra-aortic balloon pump, extensive use of electrocautery, and antibiotic administration [8, 11]. Some risk factors can not only increase the SWI incidence but also lead to the wound that is hard to heal.

Old people are the main population of thoracotomy [16]. Age is an independent risk factor for surgical incision infection. The risk of wound infection is directly proportional to age. Affected by poor tissue regeneration ability and existing systemic diseases, the old people have poor wound healing and are more prone to infection [17, 18]. According to the range of body mass index specified by the World Health Organization (WHO), obesity refers to BMI > 30 kg / m^2^, and China’s standard for obesity is BMI > 28 kg / m^2^. Engelman et al. [19] reported that obese patient with BMI > 30 kg / m^2^ were more likely to develop sternal incision infection and great saphenous vein incision infection than those with BMI < 30 kg/m^2^. The mechanism is adipocyte hypertrophy, cellular hypoxia, dysfunction of adipocytokines, increased vascular permeability, promoting immune cell infiltration into adipose tissue, releasing more inflammatory factors, and forming a vicious circle of the inflammatory response, leading to the persistence of a chronic inflammatory state [20]. In addition, thicker subcutaneous fat, larger body surface area, higher skin tension, and poor subcutaneous blood supply and lymphatic function in obese patients will also seriously affect surgical wound healing [21].

Diabetes or long-term hyperglycemia can lead to a local high glucose environment, accumulation of advanced glycation end products, microcirculatory disturbance, insufficient tissue oxygen supply, increased repair cell apoptosis, and metabolic and immune system defense dysfunction, resulting in an increased risk of infection and delayed wound healing. The absolute or relative lack of insulin in the body will form a continuous hyperglycemia state. Animal experiments showed that the sugar content of the skin tissue in diabetic mice increased, and the blood sugar level was related to skin incision. Wound healing was delayed significantly. PGP9.5IR innervation was significantly reduced, the capillary network decreased and NGF receptor expression decreased. Molecular regulation of hypoxia-related genes (HIF1A, Flt1, and KDR) is impaired, while extracellular matrix coding genes (ITGB3, TIMP1, Fn1, COL4a1) are up-regulated due to hyperglycemia and lesions [22]. Viola et al. [23] reported that in obese and diabetic patients, the number of regulatory T lymphocytes and M2 macrophages with anti-inflammatory phenotype decreased, the production of inflammatory cytokines in adipose tissue decreased, and the normal inflammation / anti-inflammatory balance was destroyed, leading to chronic inflammation. In addition, smoking and lung disease are also independent risk factors of SWI. Long-term smoking will decrease the concentration of immunoglobulin, inhibit the activity of lysozyme, reduce the number of NK cells, CD3 +, CD4 +, CD8 +, T cells, reduce the immune function, and lead to respiratory edema, increase sputum production, pulmonary infections, and even COPD [24]. Smoking, pulmonary inflammation, and COPD can cause repeated coughing, resulting in repeated friction activities at the broken end of the sternum, increasing the contact force of the fixed steel wire, and increasing the probability of sternal cracking and steel wire fracture. This situation is conducive to bacterial colonization, causing sternal necrosis and infection in the operation area, significantly increasing the probability of non-healing, and seriously affecting the quality of recovery after wound closure after debridement. Immune dysfunction is an important cause of vasculitis. Inflammation of the vascular wall will cause inflammatory cell infiltration, thickening of the vascular wall, and destruction of the vascular inner layer, narrowing or even obstruction of the vascular cavity, affecting the microcirculation and blood supply of the wound, causing a series of skin problems and the delayed wound healing for a long time. Inhibins are often used to fight immune dysfunction. Statin drugs are often used to fight immune dysfunction. However, glucocorticoid is a “double-edged sword”. It has strong immunosuppressive effects, resulting in osteoporosis and fungal infection. Fungal infection is an important factor in causing mediastinal infection [4, 25]. Patients with renal insufficiency will aggravate tissue edema, accompanied by malnutrition, anemia, and decreased immunity, which will reduce the efficiency of wound healing and aggravate the possibility of infection. It also aggravates the burden on the heart, reduces the patient’s tolerance to the surgery, interferes with the doctor’s choice of surgical strategy, reduces the effect of the debridement, and objectively increases the number of debridements [26]. Malnutrition causes additional difficulties in the debridement and reconstruction of SWI. In patients with hypoproteinemia and anemia, insufficient protein synthesis, reduction in the number of red blood cells, and insufficient oxygen-carrying capacity lead to hypoxia, slow cell regeneration, a decline in the number and function of inflammatory cells and immune cells, cause tissue edema, obstruction of granulation tissue and collagen fiber formation, and finally delay wound healing and increase the risk of infection [27]. In addition, we found in clinical practice that coagulation status also plays an important role in the occurrence and repair of SWI, which needs further clinical research. To sum up, we believe that paying attention to the adverse effects of risk factors on wound healing in advance and taking corresponding measures to intervene is the key to increase the first-stage wound healing rate and improve the efficacy of wound reconstruction.

## Clinical classification

At present, wound classification has been used to guide the treatment of SWI for a long time in clinical practice. After reviewing the literature, we found that the classification methods are various and cumbersome, and they are not uniform. This may be one of the important reasons for the current wound reconstruction methods are not uniform.

Early classification mostly used the time, depth of infection and its relationship with risk factors as the starting point to classify WSI. Early in 1984, PaiRoler et al. [28] divided SWI into three types according to the time of wound infection; Type I: incision rupture/cracking, serous exudation, sternal instability, no osteomyelitis/costochondritis within a few days (7 days) after the operation; Type II: purulent secretion with cellulitis, osteomyelitis, mediastinitis, exposed steel wire, and positive bacterial culture within a few weeks (2–6 weeks) after the operation; Type III: chronic sinus and chronic osteomyelitis are formed within months/years (6 weeks-6 years) after the operation, but mediastinitis is rare (Table 1). In order to further refine the diagnosis of DSWI and improve the treatment effect, El Oakley et al. [29] proposed a new classification and gave corresponding treatment suggestions in 1996. On the basis of the original DSWI classification, they were classified into four new subtypes based on the time of first presentation, the presence or absence of risk factors, and whether previous attempts at treating the condition have failed (Table 2). Mekontso et al. [6] divided DSWI into two types according to the onset time, Early-onset: the onset time is less than 14 days; Late-onset: more than 14 days. Gao et al. [30] In view of the complexity of SWI domestically and internationally, and no relevant reports of uninfected SWI were found, divided into three types according to whether it was infected or not, Type I: Unhealing Wound, no infection; Type IIA: Unhealing Wound, shallow infection (extraperiosteal); Type IIB: deep infection (mediastinal infection, osteomyelitis); Type III: deep sinus with localized osteomyelitis and mediastinal foreign body.

**Table 1:** Classification of postoperative stages based on surgical wound infection process proposed by PaiRolero

Classification	Postoperative stage in which infection occurs
Type I	In the first week (Sternal instability, no osteomyelitis/costochondritis)
Type II	Between 2nd to 6th weeks (cellulitis, osteomyelitis, mediastinitis, exposed wire, positive bacterial culture)
Type III	After 6th weeks to years (formation of chronic sinus tract or chronic osteomyelitis)

**Table 2:** Classification reported in 1996 by El Oakley, based on postoperative period of the infectious process and the presence of clinical risk factor

Classification	Description	Treatment strategy
Type I	DSWI (Mediastinitis) present in up to 2 weeks after the operation in the absence of risk factors	Thorough debridement and mediastinal lavage
Type II	DSWI (Mediastinitis) present in 2 to 6 weeks after surgery in the absence of risk factors	
Type IIIA	DSWI (Mediastinitis) type I in the presence of one or more risk factors	Early plastic surgery
Type IIIB	DSWI (Mediastinitis) type II in the presence of one or more risk factors	
Type IVA	DSWI (Mediastinitis) type I, II or III after treatment failure	Delayed closure of muscle or omental flap after wound debridement
Type IVB	DSWI (Mediastinitis) type I, II or III after failure of one or more treatments	
Type V	DSWI (Mediastinitis) present for the first time after 6 weeks post-operatively	Wound debridement, sternal resection, removal of exposed

Jones et al. [31] reported the first classification based on the affected anatomical structure, superficial to deep, looking at sternal stability and the presence of septicemia, and advocated single-stage debridement and closure to reduce the number and time of treatment (Table 3). Greig et al. [32] recognized that when the wound extends below the attachment point of the lower edge of the pectoralis major muscle, it is more difficult to reconstruct the lower part. In order to facilitate the treatment and reconstruction of DSWI, according to the lower margin of the pectoralis major muscle and indicating the type of reconstruction necessary for the management of deep sternal infection and dehiscence (Table 4). Rupprecht and Schmid et al. [33] classified DSWI into 3 types according to the degree of infection and sternal damage, and recommend appropriate treatment options.(Table 5). Van Wingerden proposed a classification of post-sternotomy mediastinitis, looking mainly at sternal stability, sternal bone viability, and stock, including management for the first time [34]. Based on meta-analysis and evidence-based reconstructive procedures, the authors summarized different treatment proposals and divided them into different subtypes. For example, Type I supports wound treatment through the application of negative pressure wound treatment (NPWT). In Type II, IIa can directly seal the wound without conservative treatment; IIb requires delayed closure. IIIa uses steel wire or steel plate to seal the sternum and is supplemented by NPWT; IIIb needs to be covered with a tissue flap after sternal closure. IVa needs to be repaired with myocutaneous flap after debridement; IVb was mostly closed by greater omentum; IVb uses two methods at the same time (Table 6). Anger et al. [35] improved the previous typing method (Jones and Greig), Type I refers to skin and soft tissue infection, Type II refers to sternum and rib exposure, Type III refers to sternum and rib defect, and Type IV refers to mediastinum exposure, according to the lower edge of pectoralis major muscle as a reference, determine whether it is partial or complete relative to its vertical range, and finally determine whether its position is higher or lower (Table 7).

**Table 3:** Classification proposed by Jones in 1997 based on anatomical site plus a type including sepsis

Classification	Depth	Description
Type 1a	Superficial	Skin and subcutaneous
Type 2b	Superficial	Exposure of sutured deep fascia
Type 2a	Deep	Bone exposure, sternum with stable steel suture
Type 2b	Deep	Bone exposure, sternum with unstable steel suture
Type 3a	Deep	Necrotic bone exposure or fractured, unstable sternum, exposed heart
Type 3b	Deep	Type 2 or 3 with septicemia

**Table 4:** Classification proposed by Greig in 2007, considering the location of the wound

Classification	Site of sternal wound	Recommended flap for reconstruction
Type A	Upper half sternum	Pectoralis major
Type B	Lower half sternum	Combined pectoral is major and rectus abdomen is bipedicled flap
Type C	Whole sternum	

Note: Upper and lower parts are divided vertically according to the BDC of pectoralis major

**Table 5:** Classification according to infection and sternum damage by Rupprecht et al. in 2013

Classification	Description	Treatment strategy
Type I	Noninfectious sternal instability	It is treated by rewiring, classical or Robicsek, or plating according to sternal bone status.
Type II	Deep sternal wound infection without sternal instability	It is managed by debridement, antibiotics and either primary closure, if the wound is clean, or delayed primary after NPWT using either muscle or omental flaps.
Type III	Deep sternal wound infection with sternal instability	They recommended continuous antibiotic tube irrigation with closure of the wound or leaving the mediastinum open, packed with towels or using NPWT. Later on, soft tissue reconstruction is achieved with pectoral flaps.

**Table 6:** Classification based on Assiduous Mediastinal Sternal Debridement & Aimed Management

Classification	Sternal stability	Bone vitality and stock	Reconstruction	Phased reconstruction
Type I	Stable	Reasonable	NPWT	(class I, level B)
Type Iia	-	-	Local muscle flap*	Primary (classII, level B)
Type Iib	-	-	Muscle** or Omentum flap	Delayed (class I, level B)
Type IIIa	Unstable	Viable & sufficient	Rewiring/osteosynthesis	Primary Delayed (class Iib, level B)
Type IIIb	-	-	Rewiring/osteosynthesis and muscle** or omentum flap	Primary Delayed ^(class Iib, level B)
Type Iva	-	Necrotic & insufficient	Muscle flap	Primary Delayed (class Iib, level B)
Type Ivb	-	-	Omentum flap	(class Iib, level B)
Type Ivc	-	-	Muscle and Omentum flap	(class Iib, level B)

*Always, unilateral or bilateral pectoralis muscle advancement; ** Frequently, unilateral or bilateral pectoralis muscle advancement. Rating Scheme for the Strength of the Evidence Levels of Evidence (Level A ~ Level C). Rating Scheme for the Strength of the Recommendations (Class I ~ Class III, Class II includes two subtypes) [38]

**Table 7:** Classification based on the depth and location of surgical wounds proposed by Anger and colleagues

Classification	Affected tissues	Wound location as the vertical extension	
Type I	Skin and subcutaneous tissue	Partial	Upper/ lower
		Total	
Type II	Exposure of the sternum or ribs	Partial	Upper/ lower
		Total	
Type III	Bone loss of sternum or ribs	Partial	Upper/ lower
		Total	
Type IV	Exposed mediastinum	Partial	Upper/ lower
		Total	

According to the anatomical changes and considering the depth and location of the surgical wound, the author puts forward the classification. The boundary between the upper and lower regions is the lower edge of the pectoralis major muscle

Plastic surgeons have referred to and summarized the original classification method and Schiraldi’s new treatment process, improved the existing classification method from the perspective of plastic surgeons, and proposed a treatment plan that is convenient for plastic surgeons to repair the closure [11]. (Table 8). In addition to the above-recognized classification methods, there are also various classification methods that are based on the material of coronary artery graft, sternal stability, prognosis, basic diseases, the morphology of bone nonunion, etc [36, 37]. To sum up, we can see that the early classification focuses on the time of infection and the influence of risk factors; the later classification focuses on the damage to anatomical structures but ignores the influence of risk factors on wound healing, and some classifications only focus on the classification of DSWI, while ignoring SSWI.

**Table 8:** In 2020, plastic surgeons summarized the previous classification and management

Classification	Anatomical depth	Surgical procedure
Type I	Deep sternal wound infection reaching the sternum without sternal instability	# surgical debridement +/- NPWT followed by wound revision and direct closure or using fasciocutaneous pectoral flap
Type II	Sternal instability without infection	# debridement and primary sternal closure either standard rewiring /reinforced /plates
Type III	Deep sternal infection with sternal instability with MINOR tissue/ bone loss (< 50%)	# debridement +/- NPWT followed by primary sternal closure/direct or using fasciocutaneous pectoral flap or pectoralis muscle flap
Type IV	Deep sternal infection/mediastinitis and MAJOR bone loss (> 50%)	#debridement/NPWT/Delayed primary closure (> 72 h) In #upper sternal defects: pecto-ralis major flap # lower or whole sternum defect: Pedicled rectus abdominis or great omentum

## Management

At present, there is no standard scheme for the treatment of SWI. Clinicians can choose different treatment methods according to different classification methods, mainly including several major directions: infection control, general drainage, and wound reconstruction. Although it is impossible to unify the treatment plan for wound reconstruction, two basic principles must be followed, namely, the control of infection and the treatment of sternal instability/defect. New treatment methods have been proposed continuously, each has advantages and disadvantages.

### Eliminate infection

Regardless of the classification of treatment, wound debridement and unobstructed drainage are the primary treatment for infection control [39]. If the necrotic tissue and foreign bodies in the wound were not completely removed, the forced closure of the wound would increase the chance of wound nonunion and recurrence. The long-term effect of this adverse result would result in larger wound defect and heavier infection, thus increasing the difficulty of wound reconstruction. Wound debridement only has a certain therapeutic effect on early low-grade wounds; however, the DSWI with complex wound situations has little effect and the drainage effect is not good [4]. According to El Oakley’s classification, thorough debridement and mediastinal lavage are recommended (Antibiotics + iodophor + saline) for Type I and II, and there was little difference between the wound closure effect and the treatment effect (hospital time, success rate) of the wound closure after debridement. The use of antibiotics (> 6 weeks) and thorough wound debridement according to drug susceptibility results are the keys to the treatment of DSWI [29].

One should mind that some patients have no obvious symptoms of infection. It has been reported that most DSWI patients with PaiRolero classification I-II have typical clinical manifestations such as fever, purulent exudation, and chest pain after admission. However, most of the type III patients do not have the above-mentioned typical clinical manifestations, and the indicators such as WBC and neutrophil percentage are not abnormal [40]. Therefore, we believe that multiple bacterial cultures of wound secretions, sputum, and blood should be performed in DSWI patients after admission, regardless of whether they have obvious symptoms of infection. In their study, Zhou Dan et al. [40] found that pathogenic bacteria could not be detected in about 1/2 of the 69 patients, which was considered to be related to the routine use of broad-spectrum antibiotics to prevent infection before cardiac surgery. Atypical pathogens such as bacteria and mycoplasma are also difficult to diagnose by in vitro culture. Therefore, we believe that the empirical use of antibiotics is also necessary before the results of drug susceptibility are available. The most common bacteria in SWI are gram-positive cocci, of which Staphylococcus aureus and Staphylococcus epidermidis account for more than 60%, gram-negative bacilli account for 5% ~ 22%, in addition, some are fungi, and about 25% of patients are infected by multiple pathogens [41].

Staphylococcus aureus is the most common gram-positive bacteria, while Pseudomonas aeruginosa and Acinetobacter Bauman are the most common gram-negative bacteria. Staphylococcus aureus and Staphylococcus epidermidis are highly resistant to penicillin G and sensitive to linezolid, tigecycline, ciprofloxacin, and vancomycin [40, 42]. It has been reported that linezolid is highly recommended, which has better tissue penetration, can form effective drug concentration under the sternum and mediastinum, conduct a more excellent antibacterial effect, and has an explicit clinical effect on Gram-positive bacteria in DSWI with osteomyelitis. Vancomycin has poor tissue penetration, obvious adverse effects of renal injury, and a poor antibacterial effect against methicillin-resistant Staphylococcus aureus. The drug resistance of Pseudomonas aeruginosa increased year by year, and the drug resistance of Acinetobacter baumannii increased more seriously. The former showed high resistance to imipenem, meropenem, cefuroxime, and ceftriaxone, and the drug resistance rate to common antibiotics of the latter exceeded 70% [44, 44].

In conclusion, the author believes that the wounds of those patients with DSWI mostly have residual necrotic tissue, pus, bone wax, loose steel wire, and other foreign bodies. Before the reconstruction operation, the foreign bodies need to be removed by multiple debridement operations, and the wounds need to be continuously rinsed and drained smoothly. Otherwise, even if the wound is forcibly closed, it will be difficult to heal or form a stealth sinus, which could be resulting in aggravation of infection. Clinicians must be highly skeptical about DSWI to avoid delaying the treatments for patients, although prophylactic antibiotics before cardiac surgery can improve the host’s natural defense function, it will prolong the incubation period, resulting in delayed onset of DSWI for several months after cardiac surgery, and plastic surgeons should pay close attention. After admission to the hospital, DSWI patients are recommended to perform a daily bacterial culture of secretions for three consecutive days and should be vigilant against false-negative results of bacterial culture. Even if the bacterial culture results are temporarily negative or the drug sensitivity results are delayed, antibiotics should be empirically used to prevent infection according to the local bacterial ecology and the antibiotic policy of the institution, and then the anti-infection strategy should be changed according to the drug sensitivity results.

### Keep the drainage unobstructed

Whether it is based on the classification of infection or depth of anatomy, it is advocated that thorough debridement and irrigation drainage before wound reconstruction should be carried out [29]. Negative Pressure Wound Therapy (NPWT) has been widely used in the treatment of SWI. The material selection and operation are simple, which can effectively close the wound, promote the growth of granulation, have a high wound drainage effect, and speed up the closure of the cavity. It can be applied to sternal wound fat liquefaction and osteomyelitis wound. Compared with ordinary mediastinal tube drainage after debridement, NPWT can optimize the treatment efficiency, reduce patients’ pain and maintain a continuous “debridement-like” effect. NPWT can induce venous gradient hydrostatic pressure difference, promote blood flow, reduce local osmotic active molecules effectively, reduce tissue edema, reduce microcirculation damage, maintain the tissue blood supply, reduce residual flushing fluid and inflammatory exudation, and significantly reduce patients’ pain by stabilizing the sternum halves, shorten rehabilitation time [45–47]. The risk of secondary infection and the emergence of multidrug resistant microorganisms or the erosion of the exposed right ventricle, large blood vessels and bypass pipes resulting in fatal bleeding will seriously affect the late secondary healing effect of NPWT [11]. NPWT alone can significantly promote the healing of SSWI, but it is less effective for DSWI with osteomyelitis or mediastinal infection. NPWT combined with other treatments can achieve more satisfactory results. Hao et al. [41] retrospectively analyzed 62 cases of DSWI with sternal osteomyelitis, the sinus tract sealing time, wound healing time, compared with NPWT alone, PRP combined with NPWT has great curative effects on DSWI with sternal osteomyelitis and sinus tract, for it shortens sinus tract sealing time, wound healing time, and avoids the secondary repair surgery. There are also literature reports, PNWT only induces an inferior outcome in terms of fungal infections, treatment times, and the number of reoperations [48]. Xia et al. [49] evaluated the utility of antibiotic-loaded bone cement combined with vacuum sealing drainage on DSWI, all patients’ healing wounds were first-stage healing without complications and reoperation. Federico et al. [50] found that in a high-risk patient population, the incidence of adverse events in the treatment of DSWI with NPWT and pectoralis major muscle flap was lower than that in the treatment of skin flap reconstruction alone. And preoperative NPWT makes reconstructive surgery easier and faster [51].

### Wound reconstruction

**Fig. 1:**
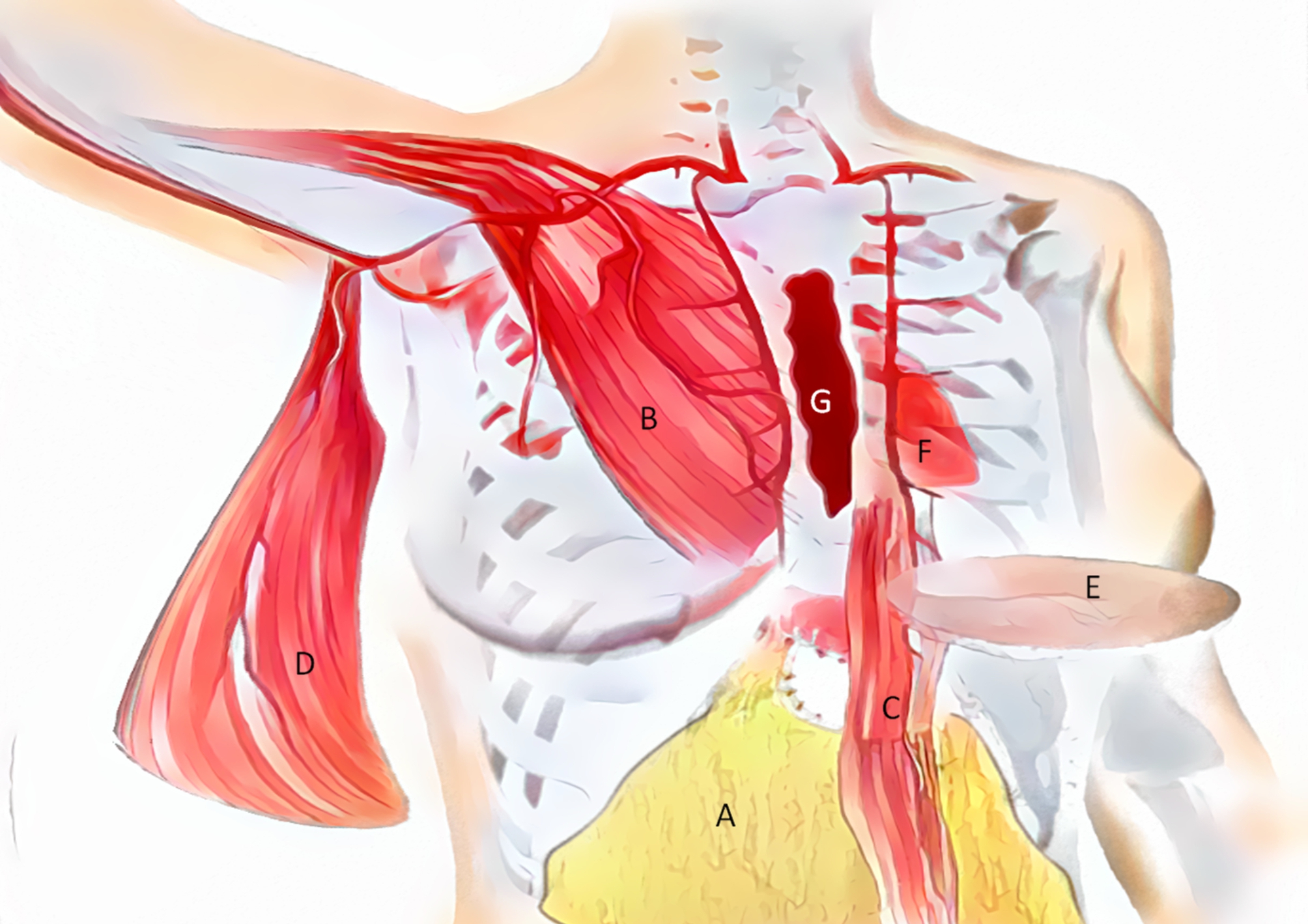
Anatomical flap location for sternal coverage (A, omentum B, pectoralis major C, rectus abdominis D, latissimus dorsi E, superior epigastric artery perforator F, heart G sternal defect)

The optimal reconstruction method of DSWI is also controversial. It is difficult to unify the results of different classifications in order to obtain a recognized and unique optimal closure method. The choice of surgical strategy ultimately depends on the wound classification, risk factors, previous surgical history, potential donor sites, and the size and tissue characteristics of the residual defect area after debridement. For DSWI with mediastinal infection, sternal instability, and sternal defect necrosis, the strategy of greater omentum transplantation or myocutaneous flap closure is the majority. The Greater omentum is the peritoneum attached to the front of abdominal organs, which can limit inflammation and prevent infection. It is rich in epidermal growth factor (EGF), promotes epithelial synthesis protein, stimulates epithelial proliferation and migration, and is rich in vascular endothelial growth factor (VEGF) to promote the reconstruction of the blood supply in ischemic tissues. Compared with traditional debridement and closed drainage, muscle or omental flap reconstruction has a complication rate and mortality of 22% and 0% respectively, while the latter has a complication rate and mortality of 92% and 33% respectively [52]. As early as the 1990s, it was reported that the success rate of greater omentum transplantation was more than 95%, and the 5-year survival rate was 82%. It was easy to operate, effectively controlled the inflammation, promoted a healing effect, and shortened the hospital stay [53]. Several articles have shown that in the case of wound infection with drug-resistant bacteria, such as wounds infected with methicillin-resistant Staphylococcus aureus, the greater omentum transplantation is more recommended, which has more advantages than muscle flap [54–56]. The disadvantage of omental transplantation is that it may cause new invasive injury, which requires training in the use of special equipment such as laparoscopy or cooperation of professional teams, and may cause complications such as epigastric hernia, bleeding, necrosis, massive exudation, and peritoneal cavity pollution, and significantly reduce the patient’s vital capacity and exercise ability [54, 57]. The musculocutaneous flap is another option besides the omental flap, for example, the unilateral or bilateral pectoralis major myocutaneous flap is still the most common reconstruction method, which is because the muscle close to the wound, as well as the function of flip or push flap. Liu et al. [58] reported that the use of unilateral or bilateral pectoralis major flap can effectively fill the DSWI residual cavity, and believed that the development of the right pectoralis major muscle is better, the muscle fiber is fuller, the blood supply is richer, and the stage I healing rate is 91.3%. In Greig’s classification, more than 50% of the severe upper sternal defect (type I) with bone loss is recommended to be closed with pectoralis major myocutaneous flap; In the lower part of the sternum or the whole sternal space (type II ~ III), the space can be filled with pedicled rectus abdominis, latissimus dorsi or greater omentum. However, the rectus abdominisflap is close to the wound and is affected by inflammation for a long time, and the internal thoracic artery has been damaged during cardiac surgery, affecting the blood supply of the flap [32]. The Cologne merheim algorithm can select the coverage strategy based on the size and depth of the wound, which means, in patients with El Oakley type IV and V DSWI, small wounds less than 6 cm were covered with unilateral or bilateral pectoral myocutaneous flaps. Unilateral pedicled pectoralis major flap is suitable for medium wounds (7 ~ 12 cm), while for large wounds (> 13 cm), it is recommended to use the left latissimus dorsi flap [59]. However, compared with pectoralis major and rectus abdominis, the latissimus dorsi flap is far away from the infection, avoids local inflammatory invasion, has a rich blood supply, and has strong anti-infectivity [60]. A recent review article compared the greater omentum transplantation with muscle flap. The results showed that the mortality from using muscle flaps was slightly higher, and the relative risk was 1.29. The greater omentum transplantation and myocutaneous flap have their own advantages and disadvantages, which need to be further demonstrated. However, a full-time and highly professional team is needed to deal with sternal wound complications, select appropriate closure or coverage, and be familiar with various types of muscle flap collection and laparoscopic greater omentum transfer. (Table 9) (Fig. 1).

**Table 9:** Comparison of wound reconstruction methods

Reconstruction mode	Advantage	Insufficient
Greater omentum transplantation	The blood supply is better than that of myocutaneous flap, the wound is smaller, the wound is more beautiful than myocutaneous flap, and the anti-infection effect is good	There are many complications and cannot provide skin and soft tissue repair. Professional team is required to cooperate with the operation.
Pectoralis major myocutaneous flap	Close to the wound, without long-distance transfer, the operation method is relatively simple, the operation time is relatively short, and the application range is wide	The substernal bone defect in the donor area cannot be used, which destroys the appearance of the chest and is not suitable for women. It is close to the wound and is invaded by inflammation
Rectus abdominis flap	It is suitable for filling the bone defect of the distal sternum	The nutrient vessel (internal abdominal thoracic artery) has been ligated during cardiac surgery, close to the wound, and attacked by inflammation, less tissue, abdominal hernia
latissimus dorsi flap	Sufficient tissue and large cutting area, the vascular pedicle is thick and long, anatomical constancy, the muscle function of the donor area is compensated by other muscles	Large trauma area, Long operation time, Changing position during operation
Platelet-rich plasma(PRP)	Hemostatic effect, release growth factors, cytokines, and bioactive proteins, good filling effect in a liquid state, analgesic effect	Platelet collection before the operation, influenced by platelet count, combined with NPWT therapy, unable to provide skin and soft tissue repair
ALBC + NPWT	Good filling and supporting effect, loadable antibiotics	Release cytotoxicity, thermogenesis, pain, it is unclear whether ALBC should be removed, delayed closure of wounds

Wounds of any type and depth are necessary to expand debridement and to remove the infection, dead bone, and foreign bodies to the greatest extent, resulting in serious wound tissue defect and sternal instability. After the debridement, the loss of soft tissue and bone and the depth of the wound are the most important factors to determine whether to close the wound in one stage and how to choose the reconstruction scheme. ( Table 9) (Fig. 1).

### New progress in treatment

Platelet-rich plasma (PRP) is an autologous plasma containing abnormal concentrations of platelets. The main component is concentrated platelets 3 ~ 10 times higher than the normal value, containing only a small amount or no white blood cells. In addition, it can also have an anti-infection ability by releasing some inflammatory inhibitory factors. It can also relieve surgical pain and neuropathic pain by regulating inflammatory response, promoting prominent regeneration, and restoring local tissue innervation [61–65]. In fact, the clinical medicine of PRP has decades of experience, widely covering orthopedics, stomatology, vascular surgery, oncology, plastic surgery and other disciplines. In recent years, it has been rapidly discovered in the field of skin and soft tissue repair and obtained good curative effect. PRP can run through the whole process of tissue repair, including hemostasis, inflammation, proliferation, and remodeling. Firstly, PRP is activated through endogenous and exogenous coagulation pathways, agglutinates into blocks, participates in the coagulation process and plays a role in hemostasis. Secondly, CXCR4 inhibits excessive inflammatory response, and bioactive proteins chemotactic mesenchymal stem cells, macrophages and fibroblasts promote inactivation, necrotic tissue clearance and tissue regeneration [66–68]. Again, platelets contain α Granules and high-density granules secrete a variety of bioactive proteins, regulate the migration and appreciation of keratinocytes, fibroblasts, and endothelial cells, promote angiogenesis, collagen synthesis, and epithelization, and then promote wound healing [69, 70]. Bielecki et al. [71] reported that PRP has an inhibitory effect on Staphylococcus aureus and Escherichia coli common in SWI. However, it was reported in the mate analysis of the effect of PRP on preventing sternal infection by Sun et al. [72], that compared with the control group, PRP can reduce the risk of postoperative nonunion of SWI by 74%, especially the therapeutic effect of SSWI, but the combined results of SWI and DSWI are heterogeneous. Hao et al. [41] found that in the treatment of sternal osteomyelitis and sinus after thoracotomy, PRP combined with NPWT can significantly improve the effect of stage I healing, reduce the number of stage II wound closure, and shorten the time of treatment and hospitalization. They also suggested that platelets should be controlled at (120 ~ 150) × 10^9^ / L, only when the platelet count in PRP reaches 1000 × 10^9^ / L or 4 ~ 7 times of whole blood platelets can achieve effective clinical results. Therefore, platelet condition is also an important factor affecting the reasonable choice of wound treatment. Antibiotic-loaded bone cement (ALBC) has a therapeutic effect on osteomyelitis. It was first reported by Klemm that bone cement cannot only conduct good mechanical support but also have a certain antibacterial effect. It has been widely used in the treatment of open fractures, osteomyelitis, and prosthesis / foreign body infection [73–75]. The advantages of ALBC in the treatment of refractory complex wounds are: ① ALBC can adjust the dosage and shape according to the size of the wound defects to better fill the residual cavity without leaving a dead cavity. The chest cavity is fixed after ALBC hardening. If necessary, it is convenient to take out and change the dressing, and the operation is simple; ② ALBC can be mixed with a variety of antibiotics according to bacterial culture and drug sensitivity results to provide mechanical support and local antibacterial. ③ Combined with NPWT, the treatment effect is obvious. However, there are relatively few public reports on ALBC for DSWI. Xia et al. [74] applied ALBC to the defect reconstruction of DSWI to solve the problem of DSWI and sternal instability and achieved a definite effect. However, it is not sure whether ALBC should be removed, or whether it will release cytotoxicity and inhibit local bone perfusion and bone remodeling, which needs further clinical research. In recent years, hyperbaric oxygen therapy (HBO_2_) has been gradually applied to the treatment of chronic and refractory wounds. It has the effects of local anti-inflammatory, down-regulating cell adhesion molecules, reducing the effect of leukocytes on endothelium, inhibiting the reproduction of anaerobic bacteria, stimulating angiogenesis, reducing edema and stimulating collagen production. Rados ł Aw et al. [76] evaluated the efficacy and effectiveness of HBO_2_ in DSWI patients. 11 DSWI patients were treated with HBO_2_ with an 80% success rate and no complications. It is considered as a valuable alternative to the treatment of recurrent refractory DSWI.

**Fig. 2:**
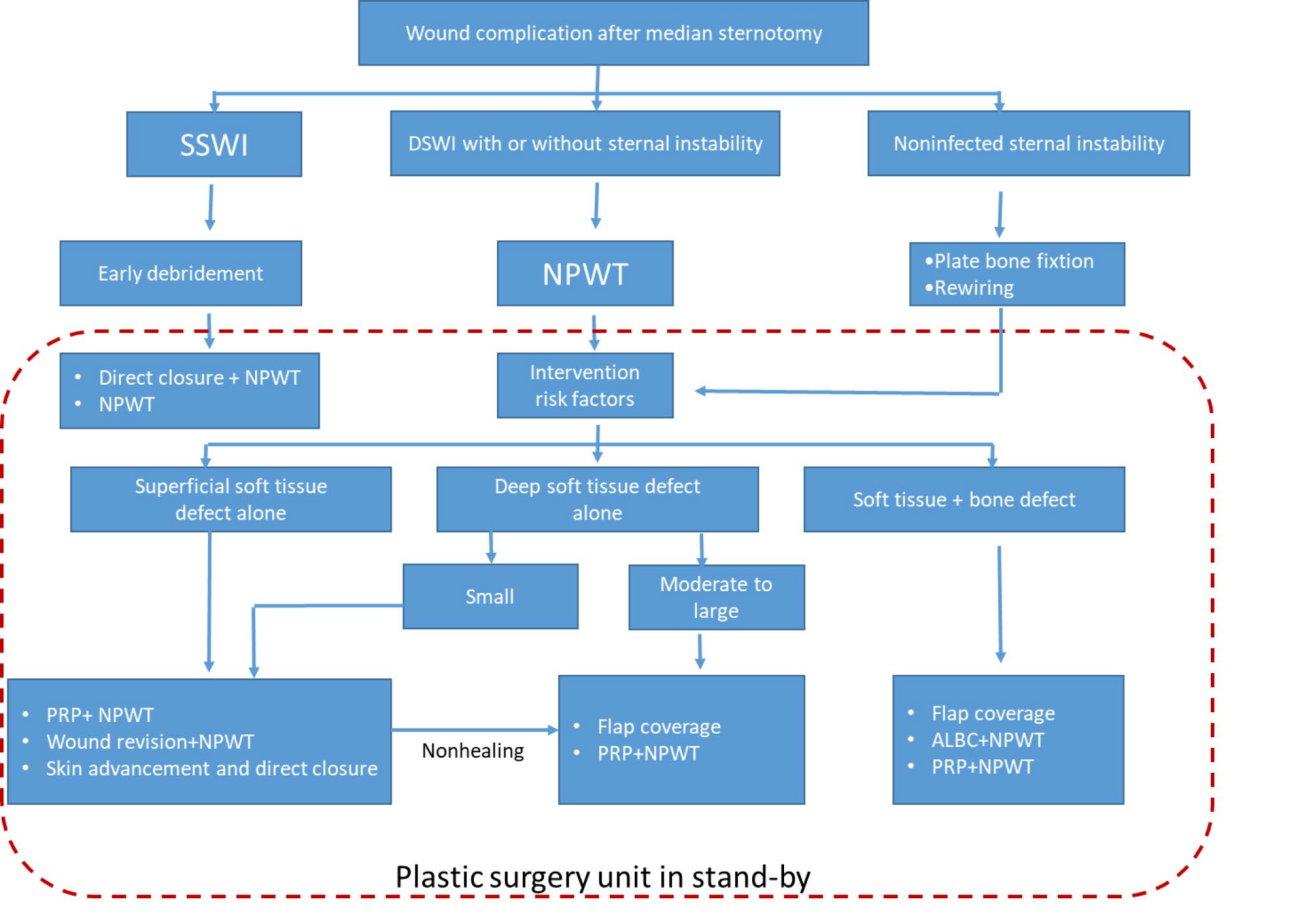
SWI acute treatment algorithm

Regardless of the type and depth of the wound, expanded debridement is required to remove infection, sequestrum, and foreign bodies to the greatest extent possible, resulting in severe wound tissue defects and sternum instability. After debridement, the amount of soft tissue and bone loss and the depth of the wound are the most important factors in deciding whether to close the wound in one stage and how to choose the reconstruction plan. We refer to and summarize the new classification proposed by plastic surgery and the practical algorithm proposed by Schiraldi, and give corresponding treatment suggestions according to different characteristics of wounds. (Fig. 2)

## Conclusion

DSWI is a potentially life-threatening complication of cardiac surgery. However, in recent years, more and more SWI patients come to the plastic surgery department for wound reconstruction. It is necessary for plastic surgeons to further understand the diagnosis, risk factors, and classification of SWI in order to select a more appropriate strategy for wound reconstruction. Accurate diagnosis is the main cornerstone in the management of this complication. Various risk factors of SWI must be taken into consideration before operating on those patients, especially the risk factors highly related to wound healing. Risk factors need to be considered together with classification to help surgeons choose more reasonable strategies for wound reconstruction. The reconstruction of complex DSWI requires the cooperation of cardiothoracic surgeons and plastic surgeons to learn from each other, so as to wait for better treatment effects.

## Data Availability

The data analyzed in this manuscript are all from the literature consulted on PubMed and CNKI, which can be found according to the content of the article.
